# ADAMTS6 suppresses tumor progression via the ERK signaling pathway and serves as a prognostic marker in human breast cancer

**DOI:** 10.18632/oncotarget.11341

**Published:** 2016-08-17

**Authors:** Yuxin Xie, Qiheng Gou, Keqi Xie, Zhu Wang, Yanping Wang, Hong Zheng

**Affiliations:** ^1^ Departments of Head and Neck and Mammary Gland Oncology, and Medical Oncology, Cancer Center, West China Hospital, Sichuan University, Chengdu, Sichuan 610041, P. R. China; ^2^ State Key Laboratory of Biotherapy/Collaborative Innovation Center of Biotherapy, West China Hospital, Sichuan University, Chengdu, Sichuan 610041, P.R. China; ^3^ Department of Anesthesiology, Mianyang Central Hospital, Mianyang, Sichuan, 621000, P.R. China; ^4^ Laboratory of Molecular Diagnosis of Cancer, West China Hospital, Sichuan University, Chengdu, Sichuan 610041, P.R. China

**Keywords:** ADAMTS6, breast cancer, tumor suppressor, ERK pathway, prognosis

## Abstract

A disintegrin and metalloproteinase with thrombospondin motifs (ADAMTS) family is involved in tumor development. However, how ADAMTS6 influences cancer remains unknown. We investigated the biological function and clinical implications of ADAMTs6 in breast cancer (BC). Its functional significance in BC cell lines was confirmed by ADAMTs6 overexpression or downregulation both *in vitro* and *in vivo* studies. Enhanced ADAMTS6 expression suppressed cell migration, invasion, and tumorigenesis, whereas knockdown promoted these characteristics. The extracellular signal-regulated kinase (ERK) pathway was partially involved in ADAMTS6-mediated inhibition of BC development, and miR-221-3p was identified as a predicted target for ADAMTS6. Results from the luciferase assay confirmed that miR-221-3p directly inhibited ADAMTS6 expression by binding its 3′-untranslated region. In addition, immunohistochemistry data from specimens from 182 BC patients showed that high ADAMTS6 expression was significantly correlated with favorable disease-free survival (DFS, *p* = 0.045). Subgroup analysis of patients with ER positive, PR positive or HER-2 negative tumors revealed that high ADAMTS6 expression more strongly extended DFS compared to low expression (*p* = 0.004, *p* = 0.009, *p* = 0.017). Multivariate analyses confirmed that ADAMTS6 expression was an independent risk factor for DFS (*p* = 0.011). Together, these data demonstrate that ADAMTS6 inhibits tumor development by regulating the ERK pathway via binding of miR-221-3p. Thus, its expression may be a potential prognostic biomarker for BC.

## INTRODUCTION

The human proteome contains 19 a disintegrin and metalloproteinase with thrombospondin motifs (ADAMTS) proteins, which are divided into 4 groups based on structural and functional similarities. These proteins share a protease domain (containing metalloproteinase and disintegrin-like modules) and a characteristic ancillary domain containing one or more thrombospondin type 1 motifs [1, 2]. Studies have demonstrated their participation in diverse functions such as collagen maturation, organogenesis, proteoglycan degradation, and inflammation [3, 4].

Although ADAMTSs have been extensively studied in the context of catalytic function, their oncogenic and tumor-protective roles were only recently revealed [5, 6]. For ADAMTS family members, metalloproteinase-related (ADAMTS1, 9) or angiogenesis-associated (ADAMTS1, 13) activity contributes to tumor development and progression [7–9], although some ADAMTSs inhibit or suppress cancer through metalloproteinase- or angiogenesis-independent activity (ADAMTS2, 12) [10, 11]. ADAMTS genes show epigenetic silencing (ADAMTS1, 8, 9, 12, 19) or genetic inactivation by DNA mutations (ADAMTS15, 18) in cancer. In addition, ADAMTS act as tumor suppressors or oncogenes, regulating matrix-degradation, physiological and pathological tissue remodeling, cell invasion and metastasis [5, 12–16]. Furthermore, ADAMTS mutation or methylation is significantly associated with improved chemotherapy sensitivity and longer overall and progression-free survival [14, 16]. Thus, the correlation between ADAMTSs and cancer needs to be explored to expand oncotherapeutic options.

ADAMTS6 is expressed in normal breast myoepithelial cells, superior cervical ganglion, trigeminal ganglion, heart, and placenta (see the BioGPS database—http://biogps.org) [1, 17]. Several studies have demonstrated that it is dysregulated in some cancers such as breast cancer (BC) [18], prolactin (PRL) tumors [19], and colorectal cancer [20]; however, the precise role of ADAMTS6 in tumor development and the underlying mechanisms is unknown. Therefore, in this study, we evaluated its biological function and relevant mechanism as well as the potential clinical applications of ADAMTS6 in BC.

## RESULTS

### ADAMTS6 expression is downregulated in human BC cell lines

**Figure 1:**
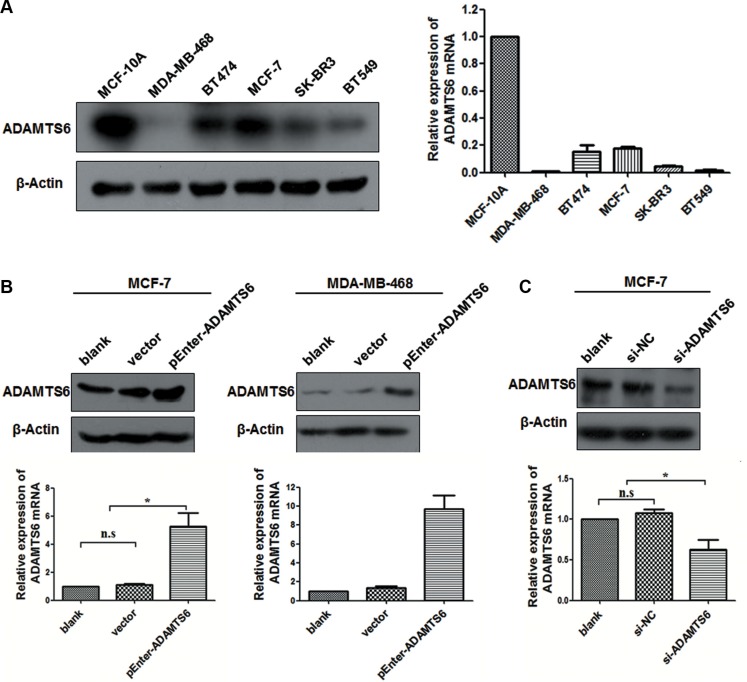
Expression and upregulation or knockdown of ADAMTS6 in BC cell lines (**A**) ADAMTS6 protein and mRNA expression (Western blotting and qPCR, respectively) in different human BC cell lines and a normal human mammary epithelial cell line (MCF-10A). (**B**) Ectopic overexpression of the ADAMTS6 gene in MCF-7 and MDA-MB-468 cell lines transfected with pEnter-ADAMTS6 (Western blotting and qPCR); Cells transfected with pEnter-vector were negative controls. (**C**) ADAMTS6 expression was downregulated by si-ADAMTS6 or scramble siRNA (si-NC) in MCF-7 cells (Western blotting and qPCR). β-actin was used as the loading control.

Baseline expression of ADAMTS6 was measured by Western blotting and quantitative real-time PCR (qPCR) in five BC cell lines, namely, MDA-MB-468, MCF-7, BT474, SK-BR3, and BT549. The human MCF-10A mammary epithelial cell line was used as the control. Compared to the control, ADAMTS6 protein and mRNA expression was downregulated in all of the experimental cell lines (Figure 1A). MCF-7 and MDA-MB-468 cells, which had the highest and lowest ADAMTS6 expression, respectively, were selected for subsequent studies. These cell lines were transfected with pEnter-ADAMTS6 plasmid overexpressing ADAMTS6 or vector (Figure 1B; *p <* 0.05); Knockdown of ADAMTS6 expression was performed with short interfering RNA (siRNA) in MCF-7 cells (Figure 1C; *p <* 0.05).

### ADAMTS6 inhibits migration, invasion, and tumorigenesis in BC cells

**Figure 2:**
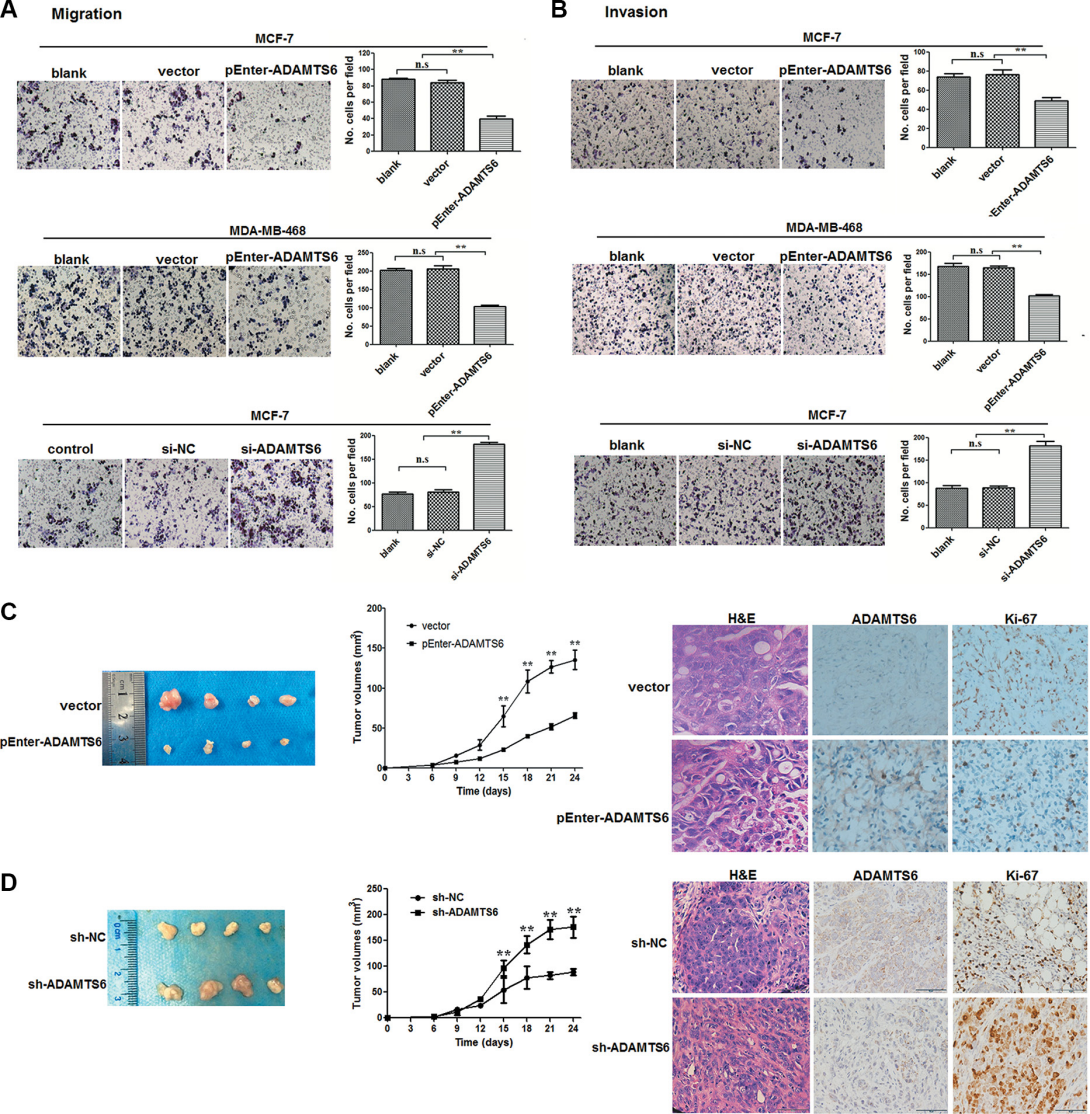
ADAMTS6 suppresses cell migration, invasion, and tumorigenesis in nude mice (**A**) Overexpression or reduction of ADAMTS6 expression inhibited or promoted, respectively, migration and (**B**) invasion in MCF-7 and MDA-MB-468 cells. Transwell chambers were used for the migration assays. Invasion assays were performed in matrigel-coated transwell chambers. Each bar represent means ± SEM of three independent experiments (100×). (**C**) MCF-7 cell-xenografted nude mice (*n* = 6/each group) were injected with vector (control) or pEnter-ADAMTS6 plasmid for 24 days. (**D**) MCF-7 cell-xenografted nude mice (*n* = 6/each group) were injected with lentivirus-mediated negative control (sh-NC) or sh-ADAMTS6, and representative tumor images (left) and tumor volume were recorded (middle) 24 days post-inoculation. Tumor sections were visualized with hematoxylin and eosin (H&E) staining and IHC for ADAMTS6 and Ki67 (right, 400×).

To understand the functional significance of ADAMTS6, we examined its effects on migration and invasion in BC cells using transwell assays. Figure 2A and 2B show that, compared with blank/vector controls, ADAMTS6 overexpression inhibited the migration and invasion of MCF-7 and MDA-MB-468 cells (*p <* 0.01). Downregulation of ADAMTS6 expression in MCF-7 cells increased migration and invasion compared to control siRNA cells (*p <* 0.01). A tumorigenesis assay showed subcutaneous tumor growth in nude mice injected with MCF-7 cells overexpressing (Figure 2C left) or lacking ADAMTS6 expression (Figure 2D left). Compared with vector controls, overexpressed or downregulated ADAMTS6 significantly repressed or enhanced tumor growth, respectively, in nude mice. Tumor volumes were significantly lower in the ADAMTS6 overexpression group and higher in the downregulated group (Figure 2C–2D, middle panel; *p <* 0.01). Immunohistochemistry (IHC) indicated that tumors from the pEnter-ADAMTS6 group had less Ki-67 indices than the controls (Figure 2C right), whereas those from the single hairpin-ADAMTS6 (sh-ADAMTS6) group had much higher Ki-67 indices (Figure 2D right).

### ADAMTS6 inhibits the epidermal growth factor/extracellular signal-regulated kinase signaling pathway

**Figure 3:**
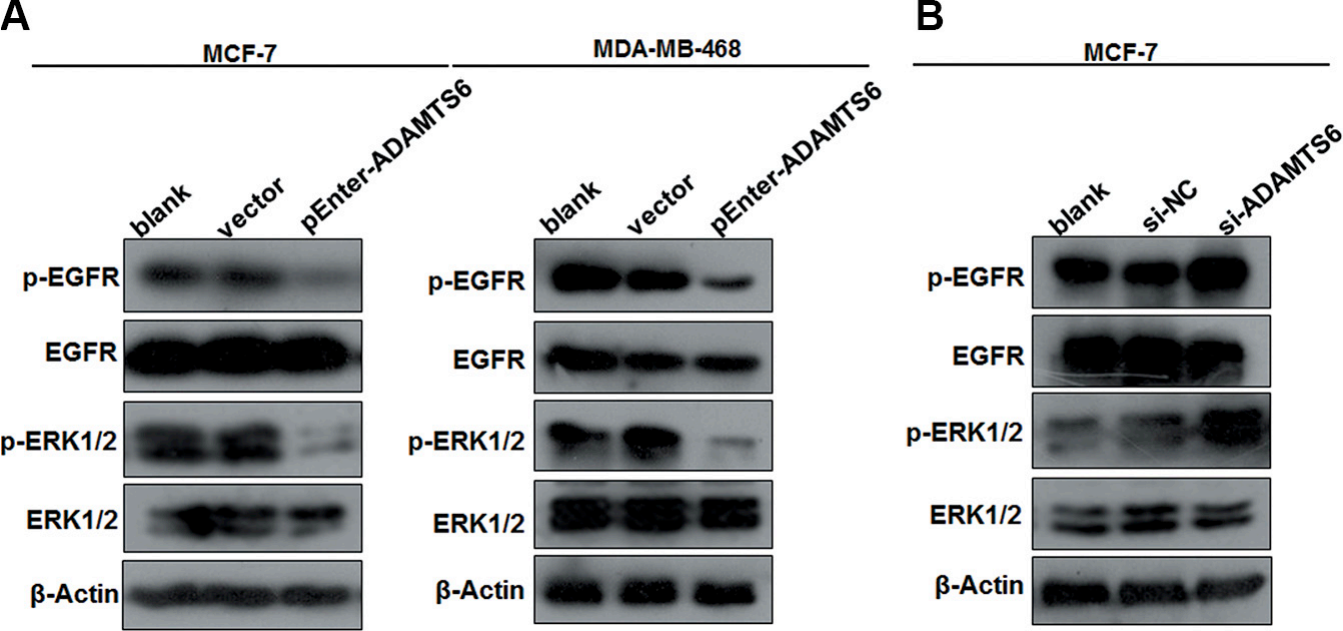
The EGFR/ERK signaling pathway is involved in ADAMTS6-mediated BC (**A**) Effects of ADAMTS6 overexpression on downstream targets of the EGFR/ERK signaling pathway (Western blotting) in MCF-7 and MDA-MB-468 cells. (**B**) Effects of ADAMTS6 knockdown on downstream targets of EGFR/ERK signaling pathway (Western blotting) in MCF-7 cells.

Metalloproteases play a role in tumorigenesis by inhibiting the extracellular signal-regulated kinase (ERK) signaling pathway [5]; therefore, we investigated the effects of ADAMTS6 on this pathway to understand the potential mechanism underlying its role in BC. ADAMTS6 overexpression dramatically decreased the expression of p-EGFR and p-ERK in MCF-7 and MDA-MB-468 cells, but did not affect total EGFR and ERK levels (Figure 3A). In contrast, knockdown of ADAMTS6 expression in MCF-7 cells significantly enhanced phosphorylation of EGFR and ERK, inducing ectopic activation of the ERK pathway (Figure 3B). Therefore, ADAMTS6 may suppress BC progression by inhibiting ERK1/2 phosphorylation.

### ADAMTS6 is a direct target of mir-221-3p

**Figure 4:**
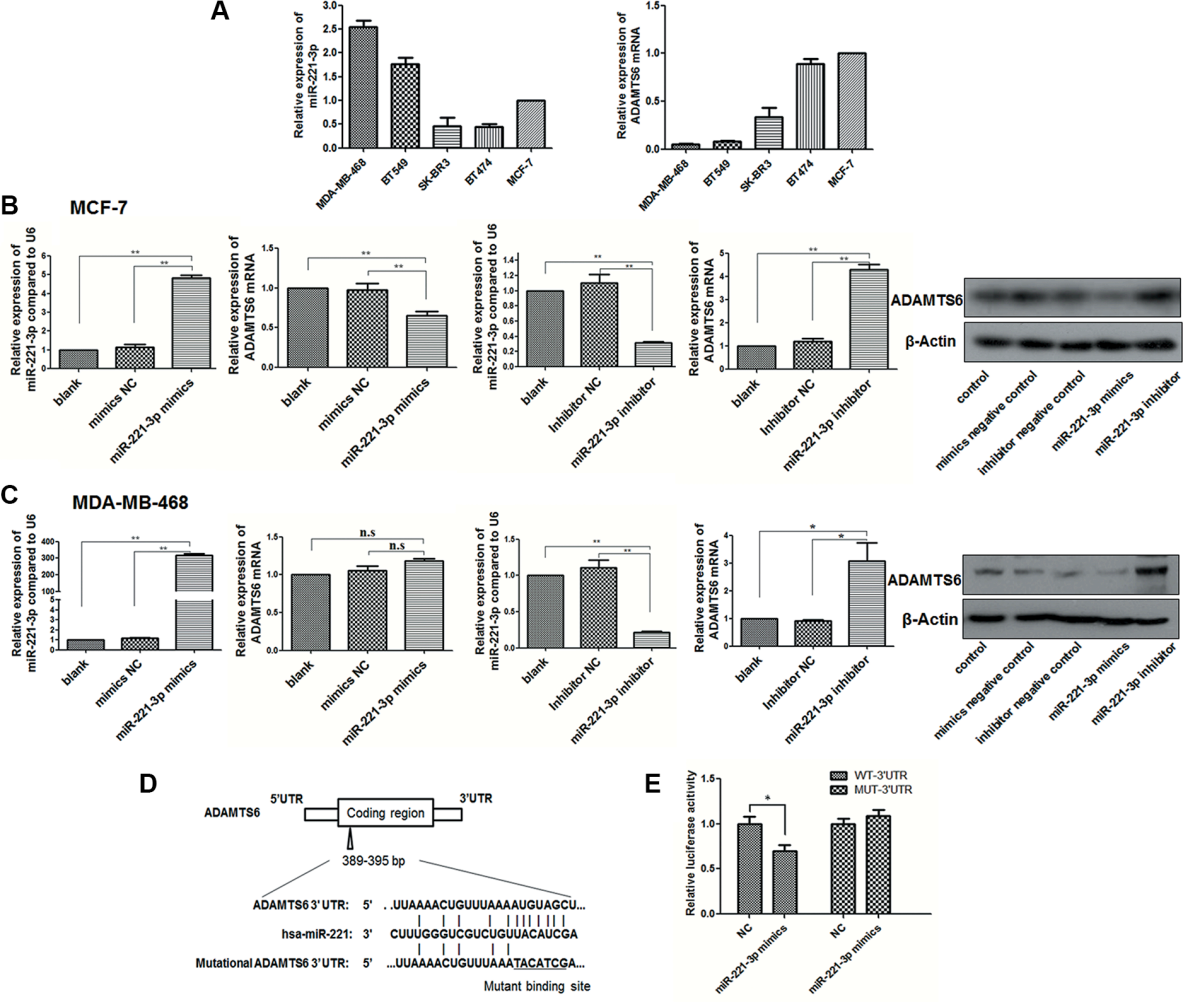
ADAMTS6 is a target of miR-221-3p (**A**) miR-221-3p and ADAMTS6 expression in five BC cell lines was measured by qPCR. (**B**) Cells were transfected with miR-221-3p mimic or inhibitor and their respective negative controls. After a 48 or 72 h incubation, cells were treated as noted in the Methods section. MiR-221-3p expression (left) corresponding to ADAMTS6 mRNA (middle) and protein expression (right) in MCF-7 and (**C**) MDA-MB-468 cells. (**D**) Schematic representation of miR-221-3p-binding sequence in the 3′-UTR of ADAMTS6 mRNA. Mutations were generated in the miR-221-binding sequence of the ADAMTS6 3′-UTR as indicated. (**E**) Luciferase reporter data with wildtype and mutated 3′-UTR of ADAMTS6 are shown after miR-221-3p overexpression.

To determine if ADAMTS6 binds any microRNAs (miRNAs), the 3′-untranslated region (UTR) of ADAMTS6 was analyzed using online databases such as TargetScan Human version 6.2 (http://www.targetscan.org). Based on the high predicted frequency, we chose 10 candidates (has-miR-144-3p, hsa-miR-18b-5p, hsa-miR-222-5p, hsa-miR-221-3p, hsa-miR-24-3p, hsa-miR-27a-5p, hsa-miR-27b-3p, hsa-miR-9-5p, hsa-miR-210-3p, hsa-miR-661) that promote BC development (confirmed from previous studies) [21–30] for verification (Supplementary Figure S1). The expression of ADAMTS6 and the candidates (Figure 4A, Supplementary Figure S2) was examined in the five BC cell lines by qPCR. The results showed that miR-221-3p and miR-210-3p expression was inversely correlated to ADAMTS6 expression in most cell lines. Next, miR-221-3p and miR-210-3p mimics or inhibitors were transfected into MCF-7 and MDA-MB-468 cells to determine if they negatively regulate ADAMTS6 expression. Upon miR-221-3p overexpression, ADAMTS6 mRNA and protein expression were reduced in MCF-7 cells (Figure 4B; *p <* 0.01). Conversely, transfection with the miR-221-3p inhibitor increased ADAMTS6 mRNA and protein expression in MCF-7 and MDA-MB-468 cell lines (Figure 4B, 4C; *p <* 0.05). However, miR-221 overexpression did not decrease ADAMTS6 expression in MDA-MB-468 cells, perhaps due to its initially low ADAMTS6 expression (Figure 4C; *p >* 0.05). No significant change in expression was observed between control and miR-210-3p mimic or inhibitor-transfected BC cells (Supplementary Figure S3). Using a luciferase assay, ectopic expression of miR-221 was shown to reduce luciferase reporter activity fused to wild-type ADAMTS6 3′-UTR, but not activity of a reporter fused to a mutant ADAMTS6 3′-UTR (Figure 4D, 4E; *p <* 0.01). Thus, the observed decrease in luciferase activity by miR-221-3p directly depends on a single binding site in the ADAMTS6 3′-UTR.

### High expression of ADAMTS6 correlates with favorable clinical outcomes

**Figure 5:**
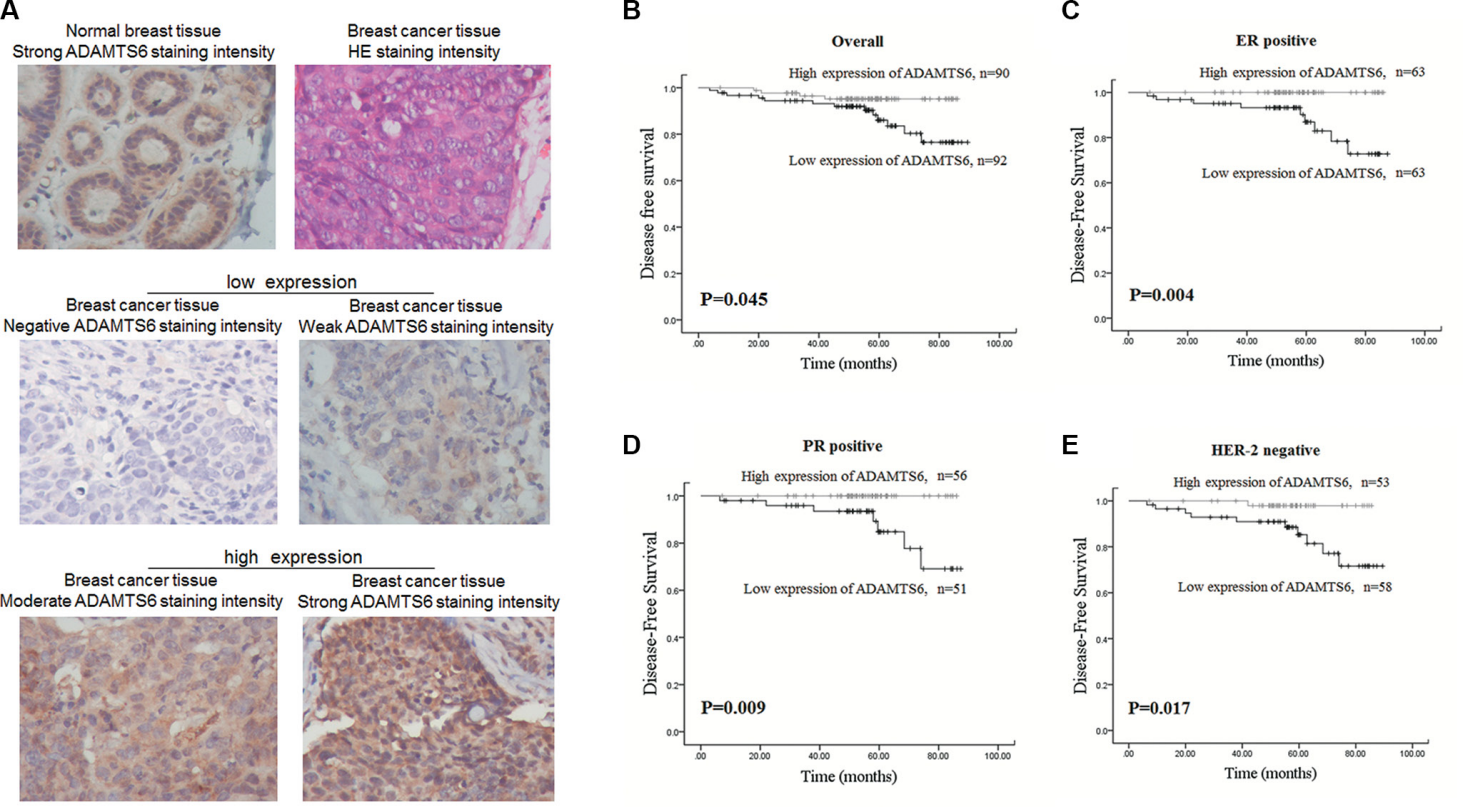
High expression of ADAMTS6 is associated with favorable prognosis of BC patients (**A**) Representative images of ADAMTS6 expression in normal mammary epithelium and BC specimens as determined by IHC. Negative and weak staining intensity of tumor tissues was defined as low ADAMTS6 expression, whereas moderate and strong staining intensity was defined as high ADAMTS6 expression (400×). (**B**) Kaplan-Meier curves for 5-year DFS according to ADAMTS6 expression in all patients. (**C**) Subgroup survival analysis according to ER-positive, (**D**) PR-positive and (**E**) HER2-negative status.

ADAMTS6 expression was evaluated in 182 primary BCs and 33 normal epithelium tissues using IHC. Among the BC samples, low expression (negative and weak staining intensity) of ADAMTS6 was confirmed in 49.6% cases (Figure 5A). There was no correlation between ADAMTS6 expression and clinicopathological status in BC patients (patient age, tumor size, stage, histological grade, or molecular subtype or lymph node metastasis) (Supplementary Table S1; *p >* 0.05). Kaplan-Meier analyses confirmed that the 5-year DFS in the group with high ADAMTS6 expression was longer than that in the low expressing group (Figure 5B; *p* = 0.045). Moreover, in the ER- or PR-positive groups, patients with high ADAMTS6 expression had a longer DFS (Figure 5C–5D; *p* = 0.004, *p* = 0.009, respectively). In addition, subgroup analysis of human epidermal growth factor receptor 2 (HER-2) status showed that patients with high ADAMTS6 expression had a significantly better DFS if they were HER2-negative (Figure 5E; *p* = 0.017). However, ER-, PR-negative and HER-2 positive group was not distinctive in this manner (Supplementary Figure S4). Importantly, multivariate analysis revealed that ADAMTS6 high expression was an independent predictive factor for better prognosis in BC patients (HR = 0.136, 95% CI = 0.029–0.636, *p* = 0.011) (Table 1).

**Table 1:** Multivariate Cox proportional analysis of disease-free survival in patients with BC

Variables	HR	95% CI	*P* value
Age(≤ 50 vs. > 50 yr)	0.922	0.285–2.981	0.892
Tumor size (≤ 2 vs. > 2 cm)	4.981	1.055–23.509	0.043*
Lymph node metastasis (yes vs. no)	2.304	0.593–8.945	0.228
Histologic grade (I/II vs. III)	0.840	0.189–3.728	0.819
ER (Negative vs. Positive)	2.676	0.493–14.535	0.254
PR (Negative vs. Positive)	1.356	0.254–7.250	0.722
HER2 (Negative vs. Positive)	2.829	0.708–11.310	0.141
Ki67 (Negative vs. Positive)	1.594	0.337–7.537	0.556
ADAMTS6 (Negative vs. Positive)	0.136	0.029–0.636	0.011*

* *p* < 0.05.

## DISCUSSION

In this study, we found decreased ADAMTS6 expression in the BC cell lines tested, indicating that its upregulation may play an important role in this disease. Thus, the effects of ADAMTS6 in BC were investigated both *in vitro* and *in vivo*. To the best of our knowledge, this is the first study to show that ADAMTS6 overexpression suppressed migration and invasion of BC cells and delayed progression of tumorigenesis in a nude mice model, and that ADAMTS6 knockdown significantly increased cell migration, invasion, and tumorigenesis. Clinically, high expression of ADAMTS6 correlated with a better DFS and was an independent marker of patient survival. These results reveal the protective function of ADAMTS6 in BC carcinogenesis and provide insight into the possible mechanism underlying its role in this disease.

The signaling pathway or mechanism underlying the suppressive effects of ADAMTS6 in BC is unknown. However, studies have suggested that the tumor-suppressive effects of ADAMTSs are linked to the deactivation of proliferation or invasion pathways, including inhibition of the ERK pathway by ADAMTS8 [13], ADAMTS12 [31], and ADAMTS15 [32]; and of the AKT/mTOR pathway by ADAMTS9 [14]. Moreover, EGFR is a direct activator of ERK oncogenic signaling, which promotes cancer progression by stimulating cell proliferation, apoptosis, metabolism, and angiogenesis. Aberrant activation of the ERK pathway is regulated by EGFR by shedding of transmembrane precursors of heparin-binding epidermal growth factor, after which downstream signaling is activated to cause carcinogenesis by stimulating the epithelial-mesenchymal transition, migration, and angiogenesis [33, 34]. Therefore, we were interested in investigating the effects of ADAMTS6 on the activation of ERK and its upstream regulator EGFR. Our results showed that ADAMTS6 reduced the phosphorylation of ERK1/2 and EGFR, suggesting that its role in suppressing BC development is likely via the EGFR/ERK pathway. However, ADAMTSs regulate cancer in a complex manner that involves multiple mechanisms [6, 35, 36]; thus, more mechanistic studies are required to better understand the role of ADAMTS6 in human cancer.

MiRNAs are small, highly conserved, noncoding RNAs that regulate gene expression post-transcriptionally by binding to the 3′-UTR of target mRNAs to inhibit translation or induce mRNA degradation [37]. We selectively chose 10 candidate miRNAs that were predicted to bind ADAMTS6 according to online bioinformatics databases and research articles. A significant observation from our work is that ADAMTS6 is a direct downstream target of miR-221-3p. MiR-221-3p is associated with proliferation, telomeres, alterations of telomerase activity, avoidance of cell death, angiogenic monitoring, and support of EMT in BC [30, 38, 39]. In addition, it can induce migration and invasion by activating RAS/ERK signaling [40]. These data support our findings that ADAMTS6 binds to miR-221-3p, thereby decreasing BC tumorigenesis. However, more studies are needed to confirm the effects of miR-221-3p in ADAMTS6-mediated inhibition of BC progression. Due to the complicated and dynamic correlation between a given miRNA and its target, additional upstream miRNAs should be identified.

ADAMTS6 expression was significantly associated with the 5-year DFS of BC patients in our study, but did not correlate with patient age, tumor size, histological grade, molecular subtype, or lymph node metastasis. The significant association of ADAMTS6 expression with survival suggests that patients with ER-positive, PR-positive or HER2-negative BC may have a favorable prognosis if ADAMTS6 expression is high. The Cox proportional hazard regression model revealed that ADAMTS6 was an independent prognostic indicator of BC, suggesting that high expression directly improves DFS in these patients. The fact that ADAMTS6 independently predicts favorable outcomes provides additional evidence for the tumor suppressor function of ADAMTS6 in BC.

**Figure 6:**
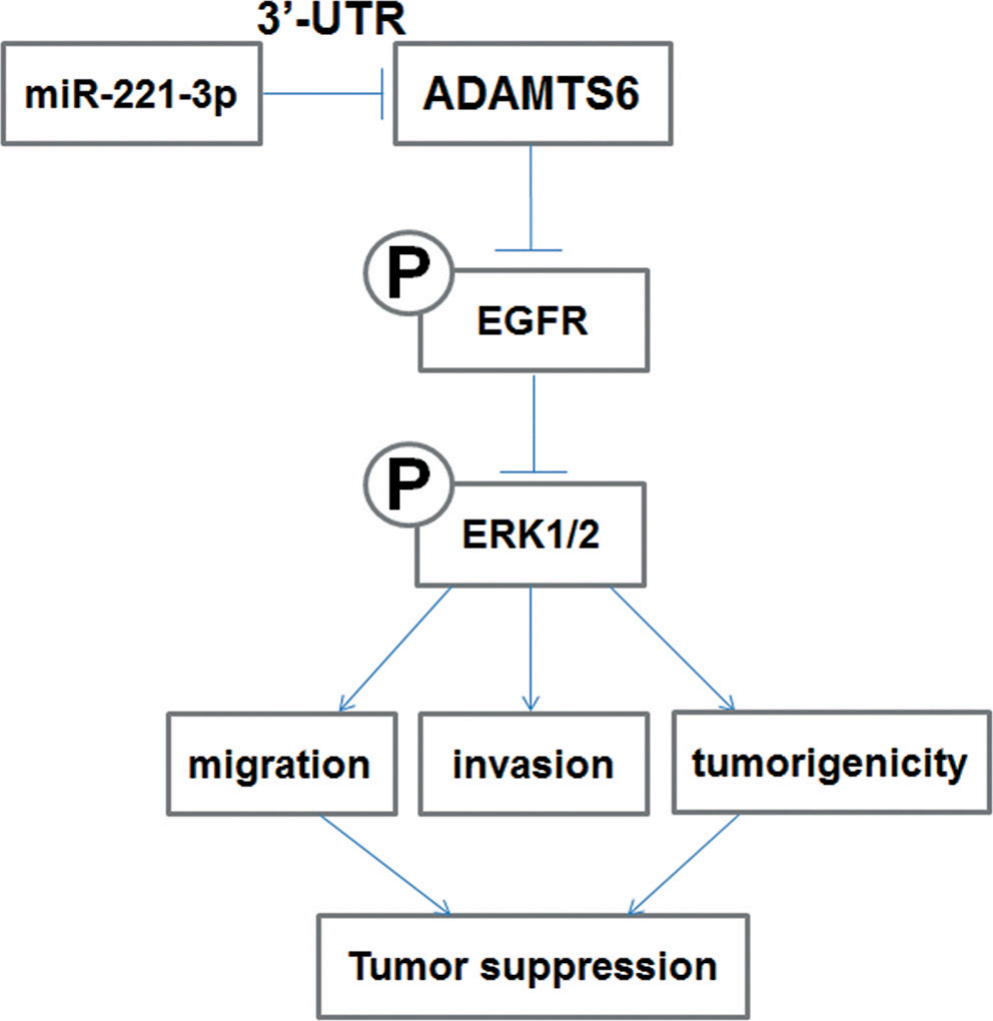
Schematic diagram illustrating the proposed ADAMTS6-mediated inhibition of the ERK1/2 signaling pathway and its role in BC cell migration, invasion, and tumorigenesis

In conclusion, we demonstrated that ADAMTS6 suppresses tumorigenesis by inhibiting cells migration and invasion *in vitro* and tumor growth *in vivo* via the ERK pathway, thereby demonstrating its protective function in BC (Figure 6). Thus, ADAMTS6 may be a prognostic biomarker and therapeutic target in BC.

## MATERIALS AND METHODS

### Cell lines

Five breast cancer cell lines (MDA-MB-468, MCF-7, BT474, SK-BR3, and BT549) were obtained from American Type Culture Collection (ATCC) (Rockville, MD). The noncancerous human mammary epithelial cell line MCF-10A was purchased from Bioleaf Biotech (Shanghai, China). They were cultured in Dulbecco's modified Eagle's medium or RPMI-1640 medium (Hyclone, Waltham, MA), respectively, according to the recommended culture method. All media were supplemented with 10% fetal bovine serum and 1% penicillin-streptomycin. All cells were grown at 37°C in an atmosphere of 5% CO_2_.

### Transfection and RNAi

MCF-7 and MDA-MB-468 cells were transfected with pEnter-ADAMTS6 or pEnter empty vector plasmid (Vigene Biosciences Inc., Shandong, China). pEnter-ADAMTS6 plasmid encodes full-length human ADAMTS6 complementary DNA (NM_197941), which was sequence-verified. Pools of stably expressing cells were generated by selection with puromycin (0.5–1 μg/ml). ADAMTS6 siRNA and siRNA negative control (si-NC) were purchased from RiboBio Company (Guangzhou, China). For GV112 lentivirus-mediated silencing of ADAMTS6, recombinant lentiviruses expressing ADAMTS6 shRNA or negative control shRNA (sh-ADAMTS6 or sh-NC) were produced by Genechem Company (Shanghai, China). The efficiency of infection was determined and shown in Supplementary Figure S5. MiR-221-3p/miR-210-3p mimic/negative control and inhibitor/negative control were purchased from GeneCopoeia (Rockville, MD). Cells were transfected with these oligonucleotides using Lipofectamine 2000 and transfection medium Opti-MEM I (Invitrogen, Carlsbad, CA, USA) according to the manufacturer's instructions. The sequences of the abovementioned RNA and DNA oligonucleotides are listed in Supplementary Table S2.

### Patients and specimens

More than 9,000 patients were registered in the Breast Cancer Information Management System of West China Hospital, Sichuan University. The breast cancer patients were recruited after excluding those who failed follow-up, underwent neoadjuvant chemotherapy, lacked complete clinical information or unable to provide a sufficient amount of tumor tissue sample in West China Hospital, Sichuan University from January 2008 to December 2012. This study was finally conducted on a total of 182 archived, formalin-fixed paraffin-embedded human breast cancer specimens. Tumor was staged according to the tumor size, lymph node involvement and distant metastasis (pTNM) classification system. Comprehensive postoperative treatment (chemotherapy, radiation therapy, endocrine therapy and targeted therapy) were administered according to NCCN guidelines [41, 42]. Patients were being followed up regularly and the median follow-up duration since the time of diagnosis was 60 months (ranged 3.7 to 89.4 months).

### IHC

All of the IHC assays were performed following the manufacturer's instructions. Briefly, consecutive paraffin sections of tissue samples were prepared and incubated overnight at 4°C with primary antibodies against ADAMTS6 (dilution 1:100; Elabscience Biotechnology Co., Ltd., Wuhan, China). Then the sections were incubated with peroxidase-labeled polymer conjugated to goat anti-rabbit immunoglobulins (EnVision/HRP, Dako, Denmark). Normal breast tissues, obtained from Cancer Molecular Diagnostics Laboratory of West China Hospital, Sichuan University, served as controls. Senior pathologists were responsible for interpreting and scoring the IHC results. The staining index was scored according to staining intensity (0, no staining; 1, weak, light yellow; 2, moderate, yellow brown; 3, strong, brown) and the proportion of positive cells (0 = 0%; 1 = < 10%; 2 = < 50%; 3 = < 75%; 4 = > 76%). An ‘immunoreactive score’ was determined to be the product of the intensity and percentage of positive cells, which ranged from 0 to 12. Cases with scores of 0–3 were defined as negative (−); 4–7 as weak (+); 8–9 as moderate (++); and 10–12 as strong (+++). Cases with scores of 0–7 were defined as the low expression group and those with scores of 8–12 were the high expression group.

### RNA extraction and qPCR

Total RNA was isolated from breast cancer cells using TRIzol reagent (Invitrogen), after which qPCR was used to quantify ADAMTS6 and miR-221-3p expression. For ADAMTS6, cDNA was synthesized using the iScript^™^ cDNA Synthesis Kit, and qPCR was performed using SsoFast^™^ EvaGreen^®^ Supermix (Bio-Rad, Hercules, CA, USA), according to the manufacturer's instructions. GAPDH was used as the internal control. For miR-221-3p, cDNA was synthesized by using the All-in-OneTM miRNA First-Strand cDNA Synthesis Kit, and qPCR was performed using the All-in-OneTM miRNA qRT-PCR Detection Kit (GeneCopoeia), according to the manufacturer's instructions. Noncoding small nuclear RNA (U6) was used as the internal control. The relative expression levels were assessed using the ΔΔCt method. The primer sequences are listed in Supplementary Table S2.

### Western blot analysis and antibodies

Western blot analysis was done as previously described [43]. Briefly, total protein extracts (30–60 μg) from cells lysates were subjected to SDS–PAGE and transferred to polyvinylidene fluoride membranes (PVDF membranes; Pierce). Primary antibodies for ADAMTS6, p-EGFR (Y1086) (dilution 1:1000; Abcam, Cambridge, MA, USA), EGFR, ERK, p-ERK (Thr202/Tyr204) (dilution 1:1000; Cell Signaling Technology, Beverly, MA, USA) were used in the tests.

### *In vitro* cell migration and invasion assays

Cell migration and invasion assays were performed in a 24-well plate with Millicell cell culture inserts (8 μm pore size; Millipore, Billerica, MA, USA). For migration assays, cells (4 × 10^4^ cells/well) were placed into the upper chamber with the non-coated membrane. For invasion assays, cells (2 × 10^5^ cells/well) were placed into the upper chamber with the Matrigel-coated membrane. In both assays, cells were suspended in 200 μl RPMI 1640 serum-free media and seeded into the upper chamber. In the lower chamber, 600 μl medium supplemented with 10% fetal bovine serum was added. After incubation for 24 h or 48 h, the chambers were fixed with 4% paraformaldehyde for 20 min, and stained with hematoxylin for 15 min. Images were captured with an optical microscope.

### *In vivo* tumorigenicity

MCF-7 pEnter-ADAMTS6, sh-ADAMTS6, and their respective negative control cells were injected subcutaneously (4 × 10^6^ tumor cells in 0.2 ml phosphate-buffered saline per animal) into the right-lower flanks of 5-week-old female nude mice (Dossy Laboratory Animal Co., Ltd., Chengdu, China). All of the experiments were conducted according to the Guide for the Care and Use of Laboratory Animals. The tumor volumes of the animals were monitored and calculated with the formula: volume = 1/2 × L × W^2^, where L is length and W is width. After 24 days, the animals were sacrificed, tumors were excised for IHC, and the tumor weights of the xenografts were measured.

### Luciferase reporter assays

The fragment of 3′-UTR of human ADAMTS6 of which contained predicted target site of miR-221-3p (wild type coding region 3′-UTR) or the site-directed mutagenesis of ADAMTS6 3′-UTR (mutant coding region 3′-UTR) was amplified by PCR and cloned into the downstream of the modified pmirGlO luciferase reporter vectors (GenePharma, Shanghai, China). All construct were confirmed by DNA sequencing.

In 293T cells, co-transfection of the reporter vectors and miR-221-3p mimics or scramble microRNA (negative control) was performed using Lipofectamine 2000. Twenty-four hours after transfection, luciferase and renilla signals were measured using the Dual Luciferase Reporter Assay kit (Promega), according to the manufacturer's protocol.

### Statistical analysis

Statistical analyses were performed using SPSS 20.0 (SPSS Inc., Chicago, IL, USA) or Prism 5.0 (GraphPad Software, La Jolla, CA, USA) software. Quantitative data were performed by a two-tailed Student *t*-test, one-way analysis of variance (ANOVA) followed by Dunnett's multiple comparison posttest. Kaplan–Meier and log-rank analyses were used to assess the survival between subgroups. A Cox proportional hazards model was used to determine the independent factors of survival and recurrence based on the variables selected in univariate and multivariate analyses. Differences were considered statistically significant at *P* < 0.05, *P < 0.05 and **P < 0.01, n.s., non-significant.

## SUPPLEMENTARY MATERIALS FIGURES AND TABLES


