# Supplementary material for: ADAMTS6 suppresses tumor progression via the ERK signaling pathway and serves as a prognostic marker in human breast cancer
Source: Oncotarget. 2016 Aug 17;7(38):61273–83. doi: 10.18632/oncotarget.11341 (PMC5308650; doi:10.18632/oncotarget.11341)
Supplement: Supplementary file 1 [file oncotarget-07-61273-s001.pdf]

ADAMTS6 suppresses tumor progression via the ERK signaling pathway and serves as a prognostic marker in human breast cancer

Supplementary Materials

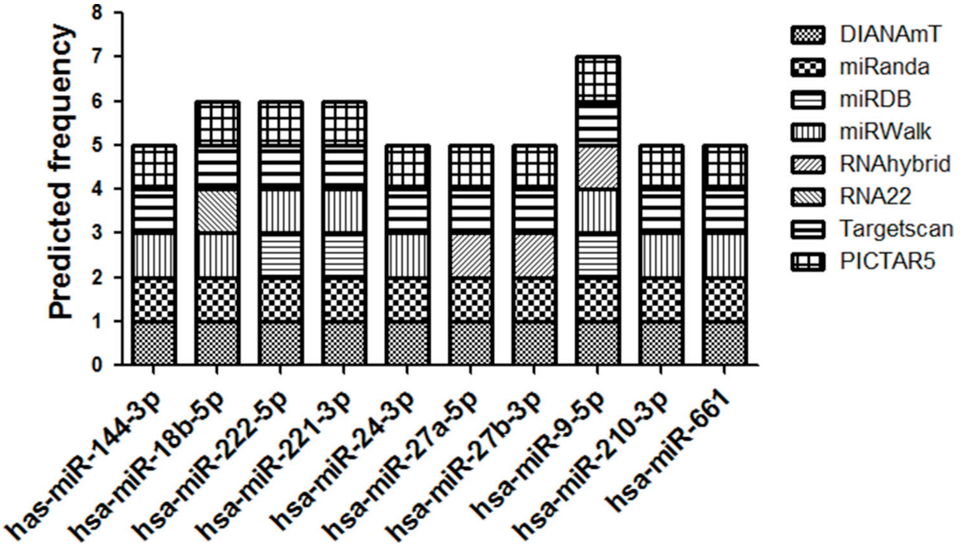

Supplementary Figure S1: Predicted frequencies of candidate miRNAs by online bioinformatics databases (DIANAmT, miRanda, miRDB, miRWalk, RNAhybrid, RNA22, Targetscan, PICTAR5) in BC.

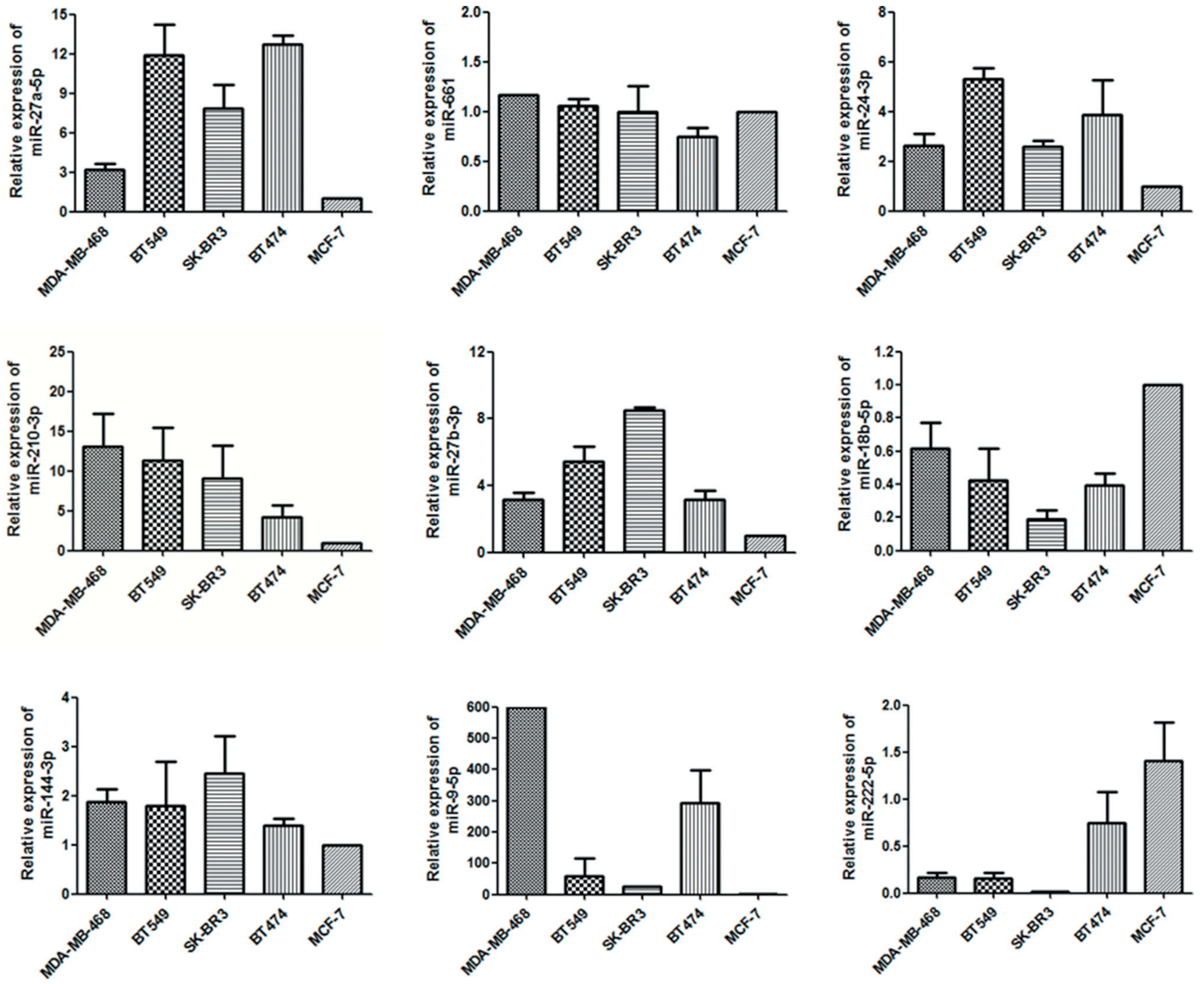

Supplementary Figure S2: The expression of candidate miRNAs was examined using qPCR in five BC cell lines.

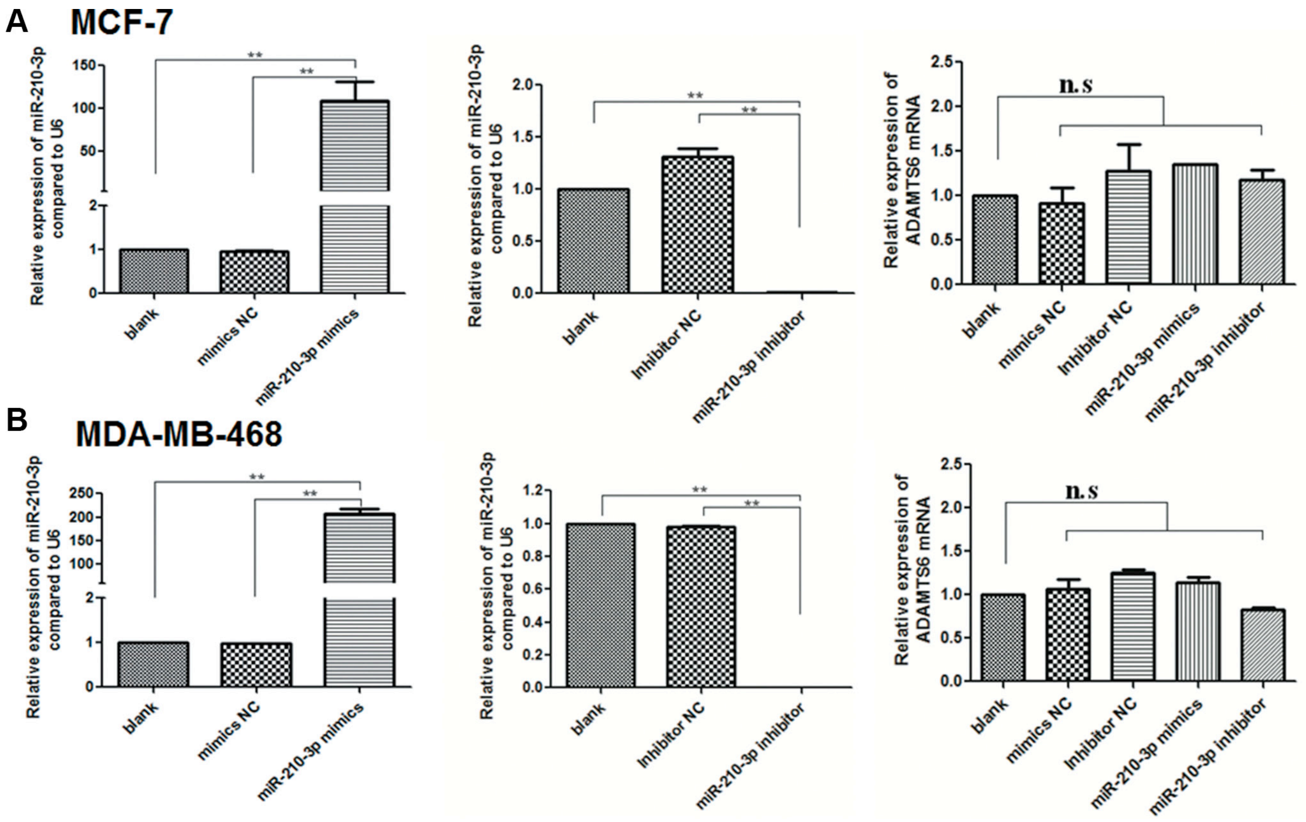

Supplementary Figure S3: Cells were transfected with miR-210-3p mimics or miR-210-3p inhibitor and their respective negative controls. After 48 h incubation, cells were treated as depicted in §Methods. MiR-210-3p expression (left and middle), corresponding ADAMTS6 mRNA expression (right) was detected in (**A**) MCF-7 and (**B**) MDA-MB-468 cells.

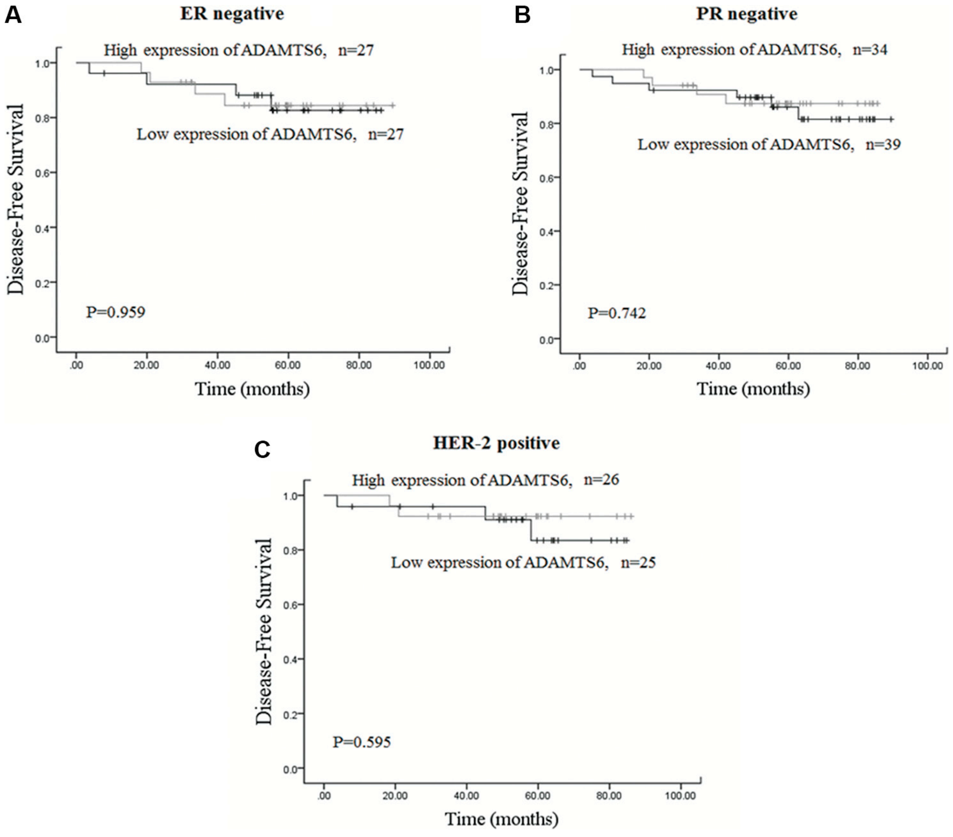

Supplementary Figure S4: Kaplan-Meier curves for 5-year DFS according to subgroup survival analysis of (A) ER-negative, (B) PR-negative and (C) HER2- positive status based on ADAMTS6 expression.

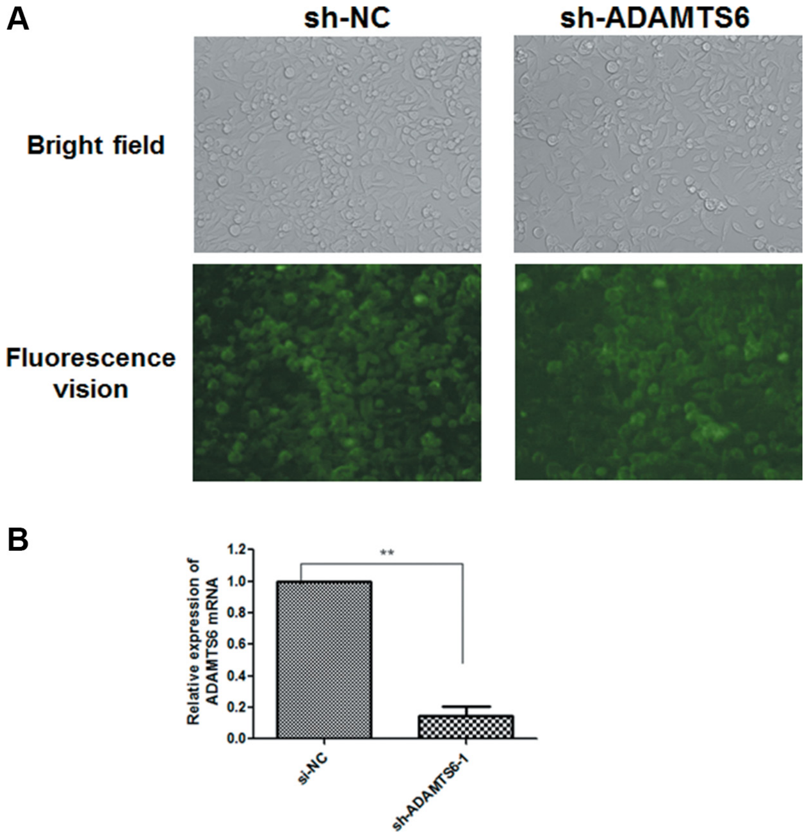

Supplementary Figure S5: (A) The transduction efficiency of the GV112 lentivirus sh-NC or sh-ADAMTS6 infected MCF-7 cells were > 90% (200× magnification). (**B**) ADAMTS6 mRNA expression was detected by qPCR.

Supplementary Table S1. Basic clinicoathological parameters of the patients and their correlation with ADAMTS6 expression in BC

Parameters	All		ADAMTS6-high		ADAMTS6-low		*P* value
	n = 182		*n* = 90 (49.45%)		*n* = 92 (50.55%)		
	No.	(%)	No.	(%)	No.	(%)	
**Age**							0.185
Median (range)	48	(27–80)	50	(31–77)	47	(27–80)	
≤ 50	102	(56.0)	46	(45.1)	56	(54.9)	
> 50	80	(40.0)	44	(55.0)	36	(45.0)	
**Tumor size (cm)**							0.367
≤ 2	77	(42.3)	35	(45.5)	42	(54.5)	
> 2–5	92	(50.5)	50	(54.3)	42	(45.7)	
> 5	13	(7.2)	5	(38.5)	8	(61.5)	
**Lymph node metastasis**							0.303
No	98	(53.8)	45	(45.9)	53	(54.1)	
Yes	84	(46.2)	45	(53.6)	39	(46.4)	
**TNM stage**							0.462
I	47	(25.8)	20	(42.6)	27	(57.4)	
II	95	(52.2)	50	(52.6)	45	(47.4)	
III	37	(20.3)	20	(54.1)	17	(45.9)	
Unknown	3	(1.6)	0	(0.0)	3	(100.0)	
**Histologic grade**							0.156
I	6	(3.3)	5	(83.3)	1	(16.7)	
II	46	(25.3)	26	(56.5)	20	(43.5)	
III	96	(52.7)	45	(46.9)	51	(53.1)	
Unknown	34	(18.7)	14	(41.2)	20	(58.8)	
**Molecular subtype**							0.561
Luminal A	19	(10.5)	12	(63.2)	7	(36.8)	
Luminal B	98	(53.8)	47	(48.0)	51	(52.0)	
Triple-negative	15	(8.2)	6	(40.0)	9	(60.0)	
HER2-type	26	(14.3)	13	(50.0)	13	(50.0)	
Unknown	24	(13.2)	12	(50.0)	12	(50.0)	
**subtyles**							1.000
Negative	54	(29.7)	27	(50.0)	27	(50.0)	
Positive	126	(69.3)	63	(50.0)	63	(50.0)	
Unknown	2	(0.1)	0	(0.0)	2	(100.0)	
**PR**							0.448
Negative	73	(40.1)	34	(37.8)	39	(43.3)	
Positive	107	(58.8)	56	(62.2)	51	(56.7)	
Unknown	2	(1.1)	1	(50.0)	1	(50.0)	
**HER-2**							0.702
Negative	111	(61.0)	53	(67.1)	58	(69.9)	
Positive	51	(28.0)	26	(32.9)	25	(30.1)	
Uncertainty	20	(11.0)	11	(55.0)	9	(45.0)	
**Ki67**							0.817
< 14%	31	(17.0)	15	(16.7)	16	(18.0)	
≥ 14%	148	(81.3)	75	(83.3)	73	(82.0)	
Unknown	3	(1.7)	0	(0.0)	3	(100.0)	

Supplementary Table S2: Sequences of RNA and DNA oigonucleotides

Name	Sense Strand/Sense Primer(5′-3′)	Antisense Strand/Antisense Primer (5′-3′)
**Primers for RT-qPCR**		
ADAMTS6	ATC ACT CGA ACT GGC AGT GG	GTC TTT GGA CAC CTC CAG CA
GAPDH	ATCATCAGCAATGCCTCC	AGTCCTTCCACGATACCAA
**microRNA mimics**		
anti-miR-221-3p		
	AGC UAC AUU GUC UGC UGG GUU UC	AAC CCA GCA GAC AAU GUA GCU UU
anti-miR-210-3p		
	CUG UGC GUG UGA CAG CGG CUGA	AGC CGC UGU CAC ACG CAC AGU U
**microRNA inhibitor**		
anti-miR-221-3p	GAA ACC CAG CAG ACA AUG UAG CU	
anti-miR-210-3p	UCA GCC GCU GUC ACA CGC ACA G	
**siRNA Duplexes**		
si-ADAMTS6		
	GGA CUA CAG UGG UUG UUU A dTdT	UAA ACA ACC ACU GUA GUC C dTdT
**shRNA Duplexes**		
sh-ADAMTS6		
Sense: GGACTACAGTGGTTGTTTA TTCAAGAGA TAAACAACCACTGTAGTCC TTTTT		
Antisense: AAAAA GGACTACAGTGGTTGTTTA TCTCTTGAA TAAACAACCACTGTAGTCC		
